# On the rotation of teleseismic seismograms based on the receiver function technique

**DOI:** 10.1007/s10950-017-9640-x

**Published:** 2017-01-24

**Authors:** M. Wilde-Piórko, M. Grycuk, M. Polkowski, M. Grad

**Affiliations:** Faculty of Physics, University of Warsaw, Pasteura 5, 02-093 Warszawa, Poland

**Keywords:** Seismic structure, Wave polarization, Sensor orientation, Automatic procedure

## Abstract

The receiver function (RF) technique is a well-established method to investigate the crustal and upper mantle structures based on three-component seismograms of teleseismic events. In the present study, we propose a modified automatic procedure to determine the back azimuth and polarization angles of a teleseismic event based on the RF technique. The method is tested for the recording of 3 permanent and 3 temporary broadband seismic stations located in the vicinity of Poland. Additionally, the analysis of Rayleigh wave polarization is conducted to show that the new procedure is not sensitive to incorrect seismometer orientation. The synthetic modelling of RF by a modified ray-tracing method for 2.5D models beneath each seismic station down to a depth of 60 km is performed to show the effectiveness of the proposed method in the calculation of RF for a complex structure with dipping layers.

**Electronic supplementary material:**

The online version of this article (doi:10.1007/s10950-017-9640-x) contains supplementary material, which is available to authorized users.

## Introduction

The receiver function (RF) technique is a well-established method to investigate the crustal and upper mantle structures based on three-component seismograms of teleseismic events (Langston 1977a; Vinnik 1977). Locally, RF provides the signature of sharp seismic discontinuities and information about the shear-wave (S-wave) velocity distribution beneath the seismic station. The initial data are broadband seismograms of teleseismic waves rotated into vertical, radial and tangential (Z, R, T) components or into a ray-parameter coordinate system (L, Q, T). After the deconvolution of the vertical component (Z or L) of the seismogram from the horizontal components (R, T or Q, T), the source, instrument and ray-path effects are removed from the seismogram. In the case of a stack of homogeneous horizontal layers, the RF is a simple scaled version of the radial component of the seismogram with the P multiples removed (Ammon 1991). Until now, researchers have focused on deconvolution methods and further interpretation and modelling of RF. Because many authors use the radial (RFR or RFQ) and tangential receiver function (RFT) for mapping dipping discontinuities and seismic anisotropy (Cassidy 1992; Zhu et al. 1995; Frederiksen and Bostock 2000; Bianchi et al. 2010), a proper rotation of seismograms is very important, especially in areas of complex structure. A modified automatic procedure for the determination of the back azimuth and polarization angles of teleseismic events based on the RF technique is proposed and tested for broadband permanent and temporary seismic stations. Additionally, three confidence testing is performed. First, the proposed procedure is tested for two simple models, one-layered and two-layered (with thin sediments), for which synthetic RFs are calculated by the *reflectivity* method (e.g. Kennett 1983). Second, an analysis of Rayleigh wave polarization is done following the method of Stachnik et al. (2012) to show that the new procedure is not sensitive to incorrect seismometer orientation. Third, synthetic modelling of RF by a modified ray-tracing method for 2.5D models beneath each seismic station down to a depth of 60 km is performed and compared with the observed back azimuth sections of RF of the presented stations.

## Tectonic settings and data

**Fig. 1:**
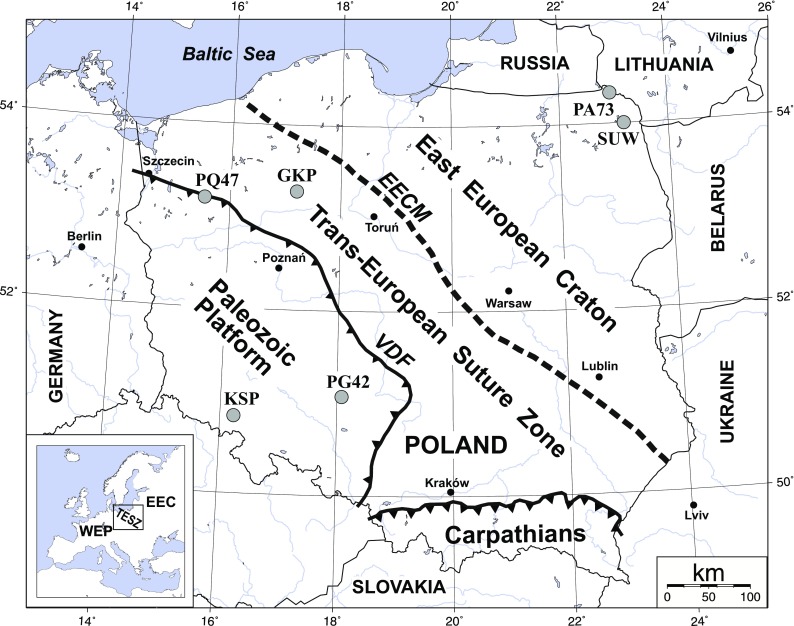
Location of the broadband seismic stations KSP, PG42, GKP, PQ47, SUW and PA73 shown against the background of simplified tectonic elements of Poland. *VDF* Variscan deformation front, *EECM* East European Craton margin

The Trans-European Suture Zone (TESZ) is a first order geotectonic unit dividing Europe into two parts (Fig. 1)—the ancient East European Craton (EEC) in the northeast and the younger Neoproterozoic-Paleozoic mobile belts (WEP) of Western Europe (Pharaoh et al. 1999). In Poland, the Paleozoic platform is characterized by a 7–12-km-thick sedimentary cover, a thin 28–34-km-thick crust with a heat flow rate of 50–70 mW/m^2^ and an age of about 450–290 Ma, while the East European Craton is characterized by a 0.5–5-km-thick sedimentary cover, a 42–47-km-thick crust with a heat flow rate <40 mW/m^2^ and an age of about 2000–800 Ma (Majorowicz et al. 2003; Grad et al. 2016).

**Fig. 2:**
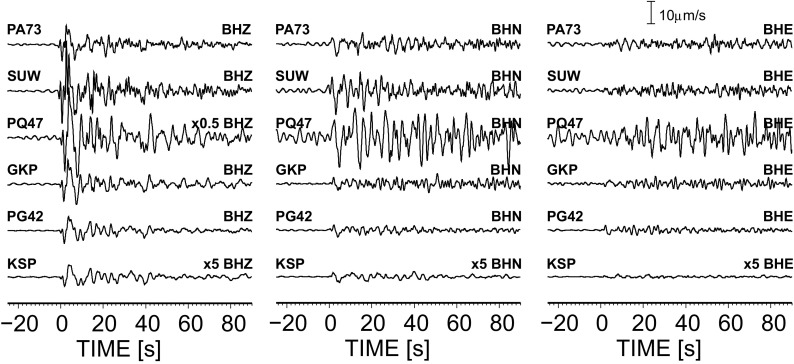
Example of teleseismic seismograms of an earthquake from the Andreanof Islands earthquake, 2007-08-15 20:22:11 (UTC), 50.322°N 177.548°W, depth 9.0 km, M 6.5. Seismograms are filtered with a band-pass Butterworth filter of corner frequencies 0.03 and 1 Hz. The amplitude scale is the same for all of the components of the seismograms (traces of KSP are increase 5 times and BHZ of PQ47 decrease 0.5 time, for a better view). Time zero refers to the theoretical P onset calculated from the *iasp91* model

The modified method of rotation of teleseismic seismograms is tested based on data from WEP, TESZ and EEC, recorded by 3 permanent broadband seismic stations (SUW, KSP and GKP) of the Polish Seismic Network and 3 temporary broadband seismic stations (PQ47, PA71, PG42) of the PASSEQ 2006–2008 experiment (Wilde-Piórko et al. 2008). The locations of the seismic stations are shown in Fig. 1 against the background of a tectonic map of Poland; their coordinates, analyzed time period and sensor type are shown in Table 1. The stations have been selected to test a rotation procedure for different tectonic units—the SUW and PA71 stations are located at the East European Craton, KSP and PG42 at the Paleozoic platform and GKP and PQ47 at the TESZ. From August 2006 to July 2008, the stations have recorded 104 P-teleseismic events with a good signal-to-noise ratio in the distance range of 30–95^o^ (for a complete list of earthquakes, see Table S1 in the electronic supplement to this article). Figure 2 shows an example of seismograms of a teleseismic P-wave from the Andreanof Islands (Aleutian Islands, Alaska) earthquake on the 15th of September 2007 with magnitude 6.5 recorded by permanent and temporary seismic stations (for KSP, the distance is 78.2^o^ and the back azimuth 9.0^o^, for PG42, 77.7^o^ and 10.1^o^, respectively).

**Table 1:** Location of seismic stations and time range of the used data set for receiver function analysis, along with the calculated orientation of the sensors

Station	Location			Sensor	Data for analysis	Number of stacked events	Orientation of station [^o^]	
	Lat. [^o^N]	Lon. [^o^E]	Elev. [m]				RF-rotation	Rayleigh waves
GKP	53.27	17.24	115	STS-2	August 2006–July 2008	63	39 ± 2	45 ± 4
KSP	50.84	16.29	353	STS-2	August 2006–July 2008	56	3 ± 1	11 ± 4
PA73	54.32	22.95	148	CMG-3TD/120	August 2006–October 2007	31	−3 ± 3	0 ± 5
PG42	51.10	18.06	180	STS-2	August 2006–July 2008	63	−21 ± 1	−16 ± 4
PQ47	53.20	15.54	95	CMG-3ESP/120	August 2006–June 2008	28	−1 ± 2	5 ± 5
SUW	54.01	23.18	152	STS-2	August 2006–January 2008	67	9 ± 1	10 ± 3

## Rotation of seismograms

Some researchers, e.g. Owens et al. (1984), calculate RF from seismograms rotated into vertical (Z), radial (R), and tangential (T) components; while others, e.g. Kind et al. (1995), use a ray-coordinates system (L, Q, T). The first approach is a one-step procedure; a back azimuth angle has to be defined to rotate the N and E components of the seismograms into R and T components. The second approach is a two-step procedure: first, a back azimuth angle is defined to rotate the N and E components into R and T; next, a polarization angle is defined to rotate the Z and R components into L and Q.

Generally, in receiver function techniques, a back azimuth angle between the north direction and a great path from the seismic station to the source of the recorded event are calculated from the known coordinates of the seismic station and the coordinates of the source taken from seismic bulletins. If we assume that a seismic sensor is properly installed, or the deviation of its orientation from true north is known, and a structure beneath a seismic station does not cause an azimuthal change of the propagation direction of waves, RF gives us a good approximation of the impulse response of the structure beneath the seismic station (Ammon 1991). Unfortunately, the seismic structure of the Earth is complex, and it often happens that the orientation of the seismometer is not precisely measured, or is unknown for ocean bottom seismometers. An incorrect choice of rotation angle will result in a wrong distribution of seismic energy between the components of RF. The orientation of seismometers can be verified by the analysis of Rayleigh wave polarization (Stachnik et al. 2012), which is not sensitive to local structure. A polarization of seismic waves can be found by the analysis of particle motion diagrams, the cross-correlation method, the smallest eigenvalue minimization method or minimizing/maximizing the energy of the components of the seismograms. These methods are more sensitive to the signal-to-noise ratio of the recorded signal because of the presence of source time functions in seismograms.

### Back azimuth angle

**Fig. 3:**
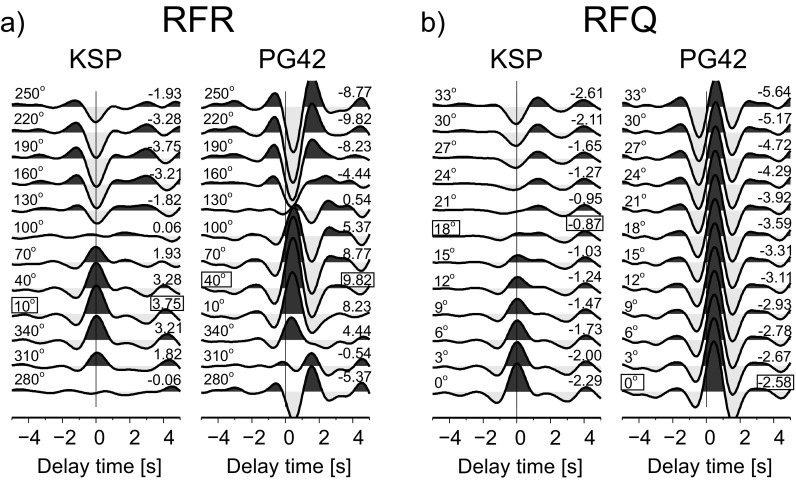
Example of the RF-rotation procedure at the KSP and PG42 stations for an earthquake in the Andreanof Islands (see Fig. 2). **a** RFR calculated from seismograms rotated from the ZNE to ZRT system every 30^o^ (*numbers at the left*); a sum of amplitudes from 0 to 1 s is shown *at the right*. **b** RFQ calculated from seismograms rotated from the ZRT to LQT system every 3^o^ (*numbers on the left*); a sum of negative amplitude from −2 to 0 s is shown *on the right*. The optimal value of the rotation angles, i.e. back azimuth and polarization angles, are marked by a *box*. Before RF calculation, the seismograms are filtered with a band-pass Butterworth filter of corner periods 2 and 10 s. Delay time zero refers to the direct P wave. The amplitude scale is the same for all RFs

**Fig. 4:**
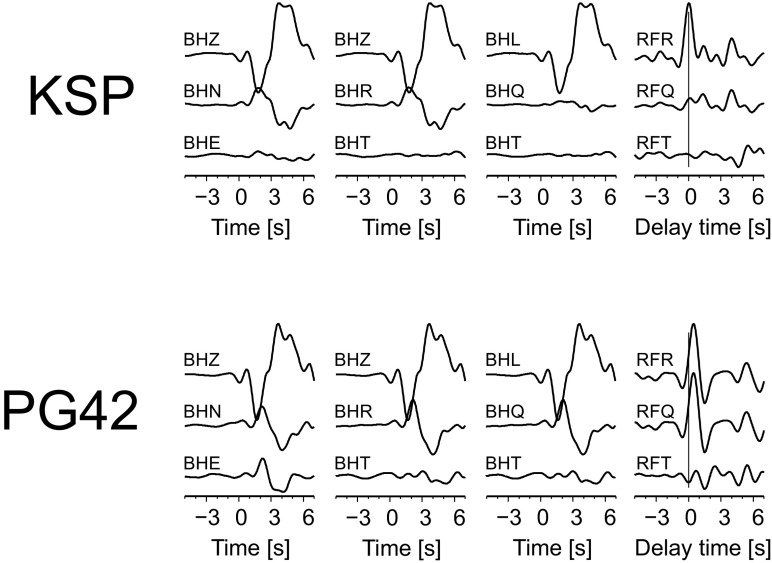
Example of the rotation of seismograms recorded by the KSP and PG42 stations for an earthquake in the Andreanof Islands with back azimuth and polarization angles found by the RF-rotation procedure (see Fig. 3). RFs are also shown. Seismograms (BHZ, BHN and BHE) are filtered with a band-pass Butterworth filter of corner frequencies 0.03 and 1 Hz. The amplitude scales are different for the seismograms and RFs. Time 0 s refers to the theoretical P onset calculated from the *iasp91* model. Delay time zero refers to the direct P wave

Previously, a method of rotation of seismogram components based on the receiver function technique was proposed by Wilde-Piórko (2015). Now, that method is improved to also work for the recordings of temporary seismic stations in the vicinity of thick sedimentary basins. First, seismograms of a teleseismic P-wave are filtered with a band-pass Butterworth filter of corner periods 2 and 10 s. Then, the N and E components are rotated with angles from 0^o^ to 360^o^, every 3^o^, and RFR is calculated for each angle. Next, the obtained RFRs were cut 5 s before and 5 s after the direct P-wave, and a mean value and a linear trend were removed to emphasize the relative change of the shape of RFR around time 0 s. The highest positive value of the amplitude of RFR at time 0 s corresponds to the back azimuth of the ray of the direct P-wave incident at the surface beneath the seismic station, not necessarily to the back azimuth of the station-event direction. The amplitudes of each RFR between time 0 and 1 s are summed to find a RFR with maximal positive amplitude in the considered time range and thereby the back azimuth of the ray of the direct P-wave. Figure 3a shows the above-described procedure for the seismograms from Fig. 2 for the KSP and PG42 stations. For the KSP station, the maximum RFR energy is observed for the back azimuth of 10^o^, while for PG42 at 40^o^. According to the catalogue, the back azimuth of KSP should be 9^o^ and for PG42 10^o^. The discrepancy for PG42 could be a result of the near-surface structure or of misorientation of the station. One could also measure the seismic amplitudes in a narrower, symmetrical time window, e.g. from −0.1 to 0.1 s, but this approach gives worse results in the presence of a high noise level and of a structure with low seismic velocities beneath the seismic station. In a longer time window, the noise will be more effectively averaged and a lack of energy at RFR at time 0 s due to a very small incidence angle of the waves travelling through the stack of horizontal layers with low seismic velocities will not result in inaccurate estimation of the direction of the ray angle. Usually, layers with low seismic velocities are thin, so the peak of the direct P-wave is superimposed at RFR by waves converted and reflected at shallow depths. Thanks to maximizing the energy of RFR at time window 0–1 s for period range 2–10 s, with the assumption that these uppermost layers have the same dip and strike like the surface, we receive a good approximation of the direction of the ray of the direct P-waves. The angle found in this way can be used later for the standard procedure of calculation of RFR for the station-event pair (Fig. 4 for the KSP and PG42 stations, and Fig. S1 in the electronic supplement for all stations).

### Polarization angle

A polarization angle can be found by maximizing/minimizing the energy on the Q and T components of the seismograms of the direct P-wave or by measuring the amplitudes of the direct P-wave on the RFR (Saul et al. 2000). In the present study, we propose to estimate a polarization angle based on RFQ components. The Z, R and T components of seismograms are filtered with a band-pass Butterworth filter of corner periods 2 and 10 s and rotated into L, Q and T components with polarization angles from 0^o^ to 45^o^; every 1^o^, RFQs are calculated. Then, the RFQs were cut 5 s before and 5 s after the direct P-wave, and a mean value and a linear trend were removed to emphasize the relative change of the shape of RFQ around time 0 s. Next, two values are calculated for each RFQ: (a) the root mean square for the time window between −2 and 0 s, to measure seismic energy, and (b) the sum of the negative amplitudes for the same time window. For a theoretical RFQ, the amplitude of the direct P-wave at time 0 s should be zero and no negative amplitude should be observed before time 0 s. The optimal polarization angle is determined when a difference of the value of the (b) parameters for successive polarization angles (starting from 0^o^) becomes negative and its absolute value increases until the (a) value reaches minimum (it is close to zero). Figure 3b shows the above-described procedure for the seismograms from Fig. 2 for the KSP and PG42 stations. For the KSP station, the difference of the (b) parameters becomes negative at an angle of 21^o^, so the optimal value of the polarization angle is 18^o^; while for PG42 it is 0^o^. Minimizing the (a) parameter only is not an optimal procedure because of the presence of noise in real data. Also, it does not work in the case of a seismic structure with thin sedimentary layers, as is the case for the PG42 station, because at time 0 s, part of the energy of the P-to-S-wave from shallow discontinuities is present at RFQ. The polarization angle found in this way can be used later for the standard procedure of calculation of RFQ for the station-event pair (Fig. 4 for the KSP and PG42 stations and Fig. S2 in the electronic supplement for all stations).

## Receiver function

**Fig. 5:**
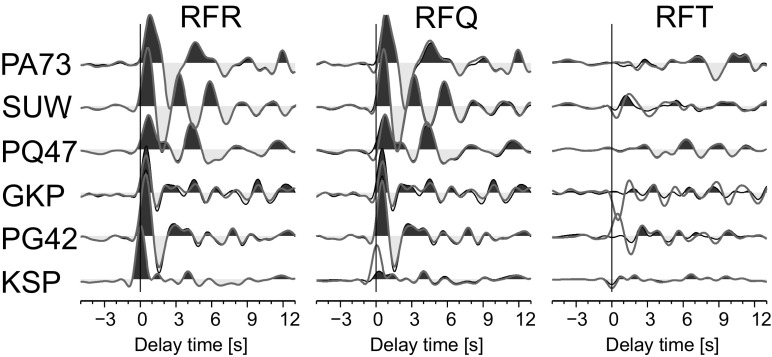
Stacked RFs for the permanent (SUW, GKP and KSP) and temporary (PA73, PQ47 and PG42) broadband seismic stations. RFs calculated based on the RF-rotation procedure are marked by *solid black lines* and *shaded areas*; RFs calculated based on the theoretical back azimuth and polarization angles are marked by *grey thick lines*. The RFs are filtered with a low-pass Butterworth filter of corner frequency 0.8 Hz. The amplitude scale is the same for all components. Delay time zero refers to the direct P wave

To test the procedure of rotation of teleseismic seismograms, RFs for 3 permanent (KSP, GKP, SUW) and 3 temporary (PG42, PQ47 and PA73) broadband stations are calculated for 104 teleseismic events, listed in Table S1 (in the electronic supplement). Seismograms are cut 100 s before and 100 s after the theoretical P onset calculated for the *iasp91* model (Kennett and Engdahl 1991) and divided by the sensitivity of the seismic station components. The back azimuth and polarization angles of each event-station pair are calculated using the procedure described above. In this study, a time-domain Wiener deconvolution and Seismic Handler package (Stammler 1993) are used for the calculation of RF. Before the deconvolution, the rotated components of the seismograms were filtered with a band-pass Butterworth filter of corner frequencies 0.03 and 1 Hz. The final RFs are move-out corrected for slowness 6.46 s/^o^ and stacked (Fig. 5—shaded area). RFs are also calculated for seismograms rotated by the theoretical back azimuth (according to USGS/NEIC PDE catalogue) and polarization angles are calculated for the horizontal half-space (Fig. 5—grey thick line). The near-surface S-wave velocities for the calculation of the theoretical polarization angle are 1.3 km/s, based on the high resolution 3D seismic model of the crust and uppermost mantle structure in Poland (Grad et al. 2016). Stacked RFs calculated with the two methods of rotation are almost the same for the PA73, SUW, PQ47 and KSP stations, and different for the GKP and PG42 stations. The amplitudes of RFT of GKP and PG42 in the case of the rotation of the seismograms by the theoretical back azimuth and polarization angles are very high, which suggest that the sensors are misoriented. Also, the S-wave near-surface velocities used for the calculation of the theoretical polarization angles for PA73 and PQ47 are too high because of the observed negative amplitudes at RFQ just before time 0 s. For KSP, the S-wave near-surface velocity is too low because at RFQ we can observe the peak at time 0 s. The reason for this is the installation of the sensor inside the mountain, in an old tunnel, so the Tertiary and Quaternary sediments present at the surface should not be taken into account for the calculation. The RFs for other stations are strongly affected by this thin layer.

## Confidence testing

**Fig. 6:**
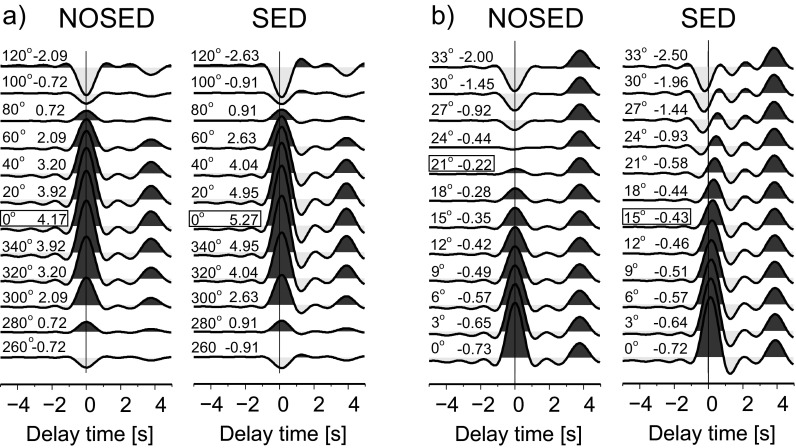
RF-rotation procedure tested for simple models NOSED and SED (see Table 2). **a** RFR calculated from seismograms rotated from the ZNE to ZRT system every 30^o^ (*first numbers on the left*); a sum of amplitudes from 0 to 1 s is shown as the *second number on the left*. **b** RFQ calculated from seismograms rotated from the ZRT to LQT system every 3^o^ (*first number on the left*); a sum of negative amplitude from −2 to 0 s is shown as the *second number on the left*. Other descriptions are similar to those in Fig. 3

Three confidence testing is performed to investigate the efficiency of the RF-rotation procedure. In the first test, the synthetic seismograms (impulse response of the structure) are calculated for simple one-layer and two-layer models (Table 2) with slowness 6.46 s/^o^ and back azimuth 0^o^ by the *reflectivity* method (Kennett 1983). Then, the back azimuth and polarization angles are sought by the RF-rotation procedure (Fig. 6). The back azimuth angle found for both models is 0^o^, as predicted, and the polarization angle is 21^o^ for the model with no sediments (NOSED) and 15^o^ with sediments (SED). From the theory, the polarization angle for the NOSED model is 23^o^ and for the SED model is 15^o^. To obtain the proper value of the polarization angle for the NOSED model, the rotation of the components should be done every 1^o^. Apart from that discrepancy, the presented procedure is successful in the case of the existence of a high contrast of seismic velocity just beneath the station.

**Table 2:** Parameters of simple flat models used for testing the RF-rotation procedure

NOSED model						SED model					
Depth [km]	Vp [km/s]	Vs [km/s]	Density [g/cm^3^]	Strike [^o^]	Dip [^o^]	Depth [km]	Vp [km/s]	Vs [km/s]	Density [g/cm^3^]	Strike [^o^]	Dip [^o^]
0.0	6.00	3.47	2.74	0.0	0.0	0.0	4.00	2.26	2.41	0.0	0.0
30.0	8.00	4.44	3.33	0.0	0.0	1.0	6.00	3.47	2.74	0.0	0.0
						30.0	8.00	4.44	3.33	0.0	0.0

**Fig. 7:**
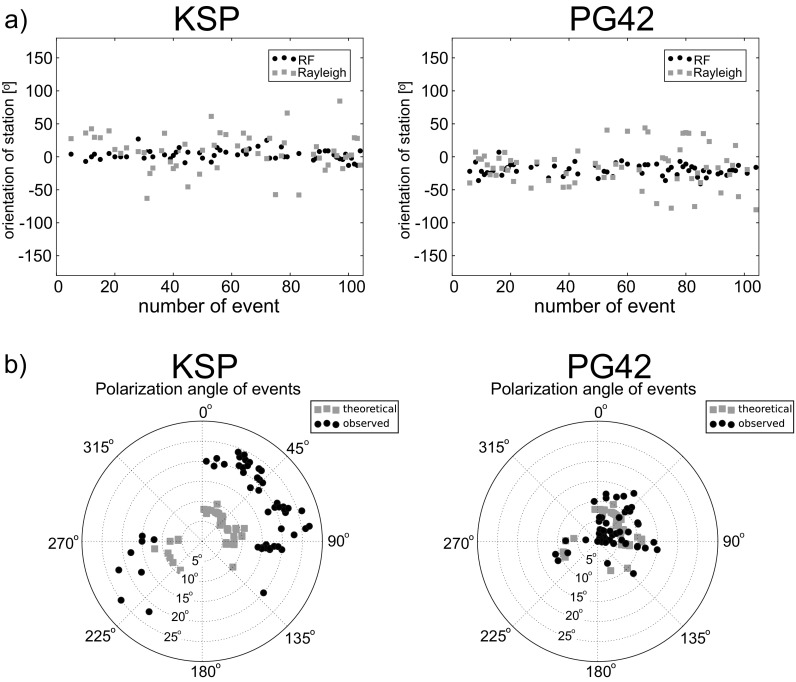
Analysis of the back azimuth and polarization angles obtained from the RF-rotation procedure. **a** Orientation of the station’s sensor calculated as the difference between the theoretical and observed back azimuth angles, based on the RF-rotation procedure (RF) and Rayleigh wave polarization analysis (Rayleigh). **b** Back-azimuthal distribution of theoretical and calculated from the RF-rotation procedure (observed) polarization angles

The analysis of Rayleigh wave polarization is conducted to independently estimate the sensor orientation of seismic stations for events with depth <100 km (events marked with * in Table S1 in the electronic supplement to this article). The applied method follows the method of Stachnik et al. (2012) with some modifications. The orientation of the sensor is determined based on Rayleigh wave polarization analysis for each event. The median value of sensor orientation of each station and its standard deviation are calculated for threshold values of the normalized cross-correlation (${C}_{z\overline{\mathrm{r}}}^{\ast }$) from 0.0 to 0.9 at every 0.1, to estimate the sensor orientation as the median of the set with the lowest standard deviation. In the present study, because of a preliminary selection of seismograms, the optimal value of the median is obtained for the whole data set of each station. The results of the analysis are shown in Fig. S3 (in the electronic supplement) and in Table 1, together with the results obtained from the RF-rotation procedure. The Rayleigh wave polarization analysis shows that the sensor of PG42 is misoriented by −16 ± 4^o^ and GKP is misoriented by 45 ± 4^o^. Similar values are obtained from the RF-rotation procedure, −21 ± 1^o^ for PG42 and 39 ± 2^o^ for GKP. Vescey et al. (2014) reported on analyzing the SKS waves that the misorientation of the GKP station is 41^o^ and the PG42 station is −22^o^. Generally, the individual values of orientation are more scattered for the Rayleigh wave polarization analysis than for the RF-rotation procedure (Fig. 7a and Fig. S3 in the electronic supplement). The situation is quite opposite for the distribution of polarization angles with back azimuth obtained from the RF-rotation procedure, comparing it with the theoretical polarization angle (Fig. 7b and Fig. S4 in the electronic supplement). This can be the effect of a more complex structure beneath the stations, and of the presence of noise in real data; however, no distinct azimuthal diversity of polarization angle has been observed.

**Fig. 8:**
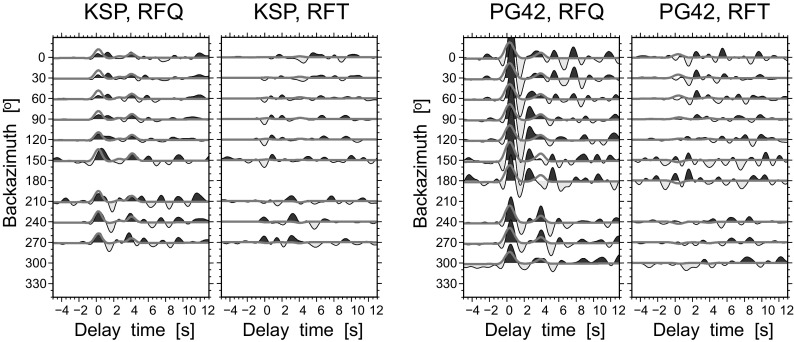
Stacked RF for the KSP and PG42 stations, sorted versus the theoretical back azimuth of events. RFs calculated based on the RF-rotation procedure are marked by *solid black lines* and *shaded areas*. RFs calculated by the modified ray-tracing method for 2.5D models (Table S2 in the electronic supplement to this article) of the structure beneath each station are marked by *grey lines*. Other descriptions are similar to those in Fig. 5

Additionally, to test the influence of the near-surface structure on the RF-rotation procedure, the synthetic RFs are calculated for 2.5D velocity models for each seismic station interpolated from the 3D high-resolution model of the crust and uppermost mantle structure in Poland (Grad et al. 2016). The 3D model provides details about the P-wave velocity distribution and geometry of the main layers of sediments (Tertiary and Quaternary, Cretaceous, Jurassic, Triassic, Permian, old Paleozoic), consolidated/crystalline crust (upper, middle and lower), and the uppermost mantle down to 60-km depth. The mean P-wave velocity in each layer and the depth of seismic discontinuities have been taken from a vertical profile beneath each seismic station; the strike and dip of the discontinuities have been calculated as a mean value from a circular area of 30 km radius at each station. The Vp/Vs ratio was assumed to be 1.8, 1.67, 1.73, 1.77 and 1.8 in the sediments, consolidated/crystalline crust (upper, middle and lower layers) and the uppermost mantle, respectively. Densities were calculated with the combined formulas of Berteussen (1977) and Gardner et al. (1974). The parameters of the 2.5D models are shown in Table S2 in the electronic supplement to this article. A modified ray-tracing method (Langston 1977b) was used to calculate the response of the structure with dipping interfaces to the incoming plane wave with fixed slowness and back azimuth. In the present paper, the original method was extended by implementation of a surface with non-zero dip and strike values. The synthetic RFs are calculated for waves directly approaching the surface (without any multiple reflection), taking into account all combination of P-to-S conversion at each discontinuity with slowness and back azimuth determined for each event. The synthetic RFs are move-out corrected for slowness 6.46 s/^o^. Then, the observed and synthetic RFs are processed in the same way: stacked in 12 bins overlapping by 30% and filtered with a double-pass low-pass Butterworth filter of corner frequency 0.8 Hz (Fig. S5 in the electronic supplement). It apparent that the values of the dip of discontinuities beneath the KSP and PG42 stations are not high enough (<5^o^) to give a visible effect on RFTs (Fig. 8). The first phase of KSP at RFQ is well modelled only for the south and southwestern back azimuth, while the P-to-S phases from the Moho discontinuity are well modelled for the north and northeastern back azimuths. The KSP station operates close to the Sudetic Marginal Fault, running from northwest to southeast, at which the sharp jump of Moho depth by 4 km is observed (Wilde-Piórko et al. 2005). As a result, the back azimuth section of RFQ for KSP is very different from the north and south back azimuth, and that diversity is not predictable by the 2.5D model. In the case of PG42, the observed near-surface effect is very strong and masks later phases. The modelled first phase of RFQ has a proper delay time, though its amplitude is too low compared with the observed one. The observed high amplitudes of RFT are not explained by 2.5D modelling using only converted waves without their multiple reflections. Additionally, for PG42, the observed high amplitudes at RFT can be the result of a pure data set (temporary station) and the complexity of the structure beneath the seismic station for KSP. Nevertheless, the first second of the observed back-azimuthal sections of RFQ are modelled well enough by the simple 2.5D modelling, which confirms the effectiveness of the RF-rotation procedure.

## Discussion

**Fig. 9:**
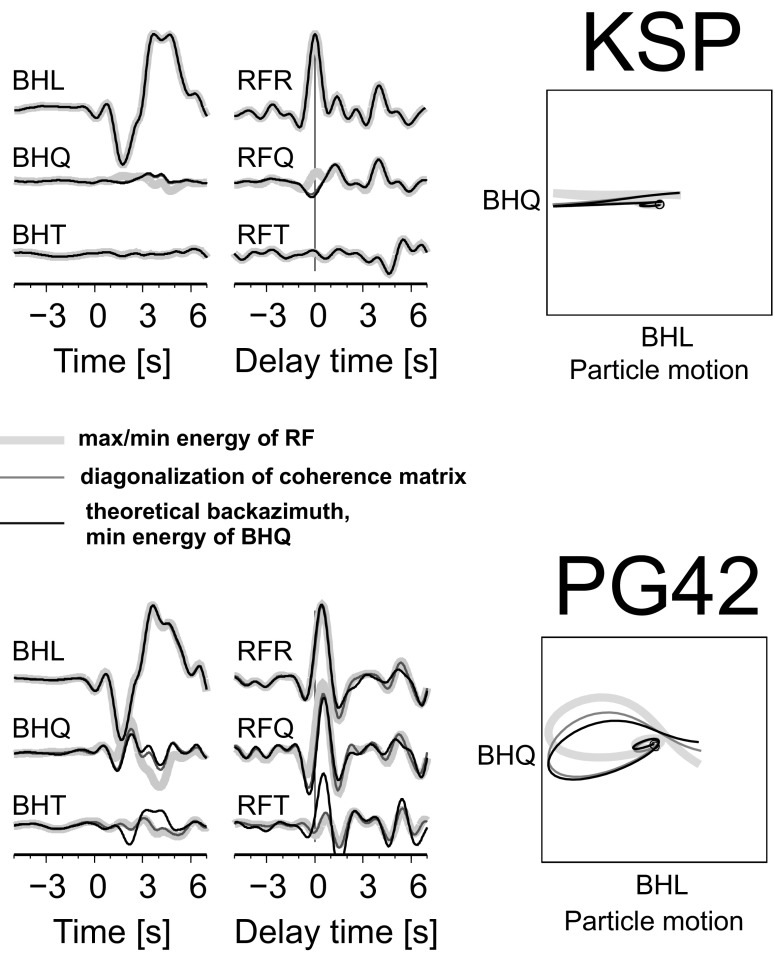
Example of the rotation of seismograms recorded by the KSP and PG42 stations for an earthquake in the Andreanof Islands with the back azimuth and polarization angles found by the RF-rotation procedure (*thick bright grey line*), with the back azimuth and polarization angles found by diagonalization of a coherence matrix of the components of the seismogram (*thin dark grey line*), and with the theoretical back azimuth angle and the polarization angle found by minimization of the amplitude/energy of the direct P-wave at BHQ (*thin black line*). RFs are also shown. Seismograms are filtered with a band-pass Butterworth filter of corner frequencies 0.03 and 1 Hz. The amplitude scales are different for seismograms and RFs. Time 0 s refers to the theoretical P onset calculated from the *iasp91* model. Delay time zero refers to the direct P wave. The corresponding particle motion diagrams of the BHL and BHQ components are also shown

In most cases, a theoretical back azimuth angle is used to rotate the Z, N and E components of a seismogram into the Z, R and T system in receiver function analysis. However, Geissler et al. (2008) reported that for stations in orogens, such as in the Alps and Carpathians, the deviations between the theoretical and observed back azimuths are significant. For such areas, they have calculated the back azimuth angles by polarization analysis of the horizontal components and the polarization angles by minimizing the energy on the Q component at the time of the P signal by computing the eigenvalues of the covariance matrix, following Kind et al. (1995). Knapmeyer-Endrun et al. (2013) also have determined the rotation angles by polarization analysis of the first onset. Kumar et al. (2006) and Kumar and Kawakatsu (2011) used theoretical back azimuth and polarization angles such that an amplitude of the P component is minimized (changed sign) at zero time; the arrival time of the reference P-wave. A search for the amplitude is automatic in a time window ±1 s due to the theoretical phase onset. We have exploited the above approaches for the rotation of seismograms of the Andreanof Islands (Aleutian Islands, Alaska) recorded by the KSP and PG42 seismic stations (Figs. 2 and 3). Figure 9 presents the L, Q and T components of a seismogram rotated by three methods: (1) the method presented in this paper, by maximizing/minimizing the energy of RF; (2) by polarization analysis (diagonalization of the coherence matrix of the components of the seismogram); (3) by using the theoretical back azimuth angle and minimizing the amplitude/energy of the direct P-wave on the Q component of the seismogram. The RFs and particle motion diagrams are also shown. For the KSP station, we have received the following back azimuth and polarization angles, respectively, for the above methods: (1) 11/19^o^, (2) 9/25^o^, (3) 9/26^o^ and for the PG42 station (1) 43/6^o^, (2) 38/19^o^, (3) 10/24^o^. The polarization analysis and RF-rotation method give a good estimation of the back azimuth angle for both stations; however, the value of the polarization angle is overestimated by methods (2) and (3), which can be seen in the particle motion diagrams (Fig. 9). Kumar and Kawakatsu (2011) have claimed that the incident angles received by their method may be erroneous, and they have discarded the events with incidence angle >52^o^. That could be the result of noise, but also, as shown in the above calculation, the result of an incorrect value of back azimuth angle. Additionally, in the presence of a thin sedimentary layer beneath the seismic station, the simple minimization of the amplitude/energy at the time of the reference wave is not an effective procedure.

## Conclusion

The RF-rotation method is automatic and effective and can be used to find the back azimuth and polarization angles of a teleseismic event recorded by permanent and temporary broadband seismic stations. In the present study, the stations are located in the Paleozoic platform deformed by Variscan orogeny (KSP, PG42), in the area of a thick sedimentary basin in TESZ (GKP, PQ47), and in the East European Craton with thin sedimentary cover and a strong contrast of seismic velocities between sediments and crystalline crust (SUW, PA73). The latter area particularly causes problems with RF calculation because of the existence of a strong reverberation of waves just beneath the seismic station. The recordings of the temporary stations are also usually strongly affected by the near-surface structure. The presented method of rotation of teleseismic seismograms based on the RF technique shows its ability to deal with such issues. Additionally, the method is not sensitive to misorientation of the seismic sensor, which is very valuable in the case of temporary campaigns. Based on the presented method, it is possible to find a sensor orientation of land and ocean seismometers (if vertical orientation is fixed, and if not, the three dimensional grid search of parameters is necessary). The sensor orientation found by the RF-rotation method is confirmed by the Rayleigh wave polarization and is previously found by SKS wave analysis.

## Electronic supplementary material

ESM 1 (PDF 1544 kb)
