# Supplementary material for: On the rotation of teleseismic seismograms based on the receiver function technique
Source: J Seismol. 2017 Jan 24;21(4):857–68. doi: 10.1007/s10950-017-9640-x (PMC5496972; doi:10.1007/s10950-017-9640-x)

Electronic Supplement to the article in Journal of Seismology

On the rotation of teleseismic seismograms based on the receiver function technique

by M. Wilde-Piórko[1], M. Grycuk, M. Polkowski, M. Grad

[1] University of Warsaw, Faculty of Physics, Pasteura 5, 02-093 Warszawa, Poland; e-mail: mwilde@igf.fuw.edu.pl

Rotation of seismograms of the Andreanof Islands earthquake and back-azimuthal sections of receiver function

In the present study, we propose a modified automatic procedure to determine the back-azimuth and polarization angles of teleseismic event based on the receiver function (RF) technique. The method is tested for the recording of 3 permanent (KSP, GKP, SUW) and 3 temporary (PG42, PQ47, PA73) broadband seismic stations located in the vicinity of Poland. Figs. S1 and S2 show an example of the rotation of the seismograms recorded by the analyzed seismic stations for an earthquake in the Andreanof Islands with the back-azimuth and polarization angle found by the RF-rotation procedure.

Additionally, the analysis of Rayleigh wave polarization is performed to calculate the orientation of the station's sensor and to compare it with the orientation calculated from the RF-rotation procedure (Fig. S3). The back-azimuthal distribution of theoretical (calculated for a horizontal half-space) and observed (calculated from the RF-rotation procedure) polarization angles are shown (Fig. S4). The parameters of the analyzed events are listed in Table S1. Stacked RFs for the analyzed seismic stations, sorted versus theoretical back-azimuth of events, are shown in Fig. S5 together with the synthetic RFs calculated by a modified ray-tracing method for 2.5D models (Table S2) of the structure beneath each station.

Table S1 Origin time and coordinates of 104 teleseismic events according to USGS/NEIC PDE Catalogue analyzed in the study. Events used for Rayleigh wave polarization analysis are marked by *.

Date y/m/d	Time h:m:s	Lat. [°]	Lon. [°]	Depth [km]	Mag.	Date y/m/d	Time h:m:s	Lat. [°]	Lon. [°]	Depth [km]	Mag.
06/08/06*	18:16:39.0	+26.12	+144.01	20	5.9	07/09/03*	16:14:53.0	+45.84	+150.06	94	6.2
06/08/11*	20:54:14.0	+2.40	+96.35	22	6.2	07/09/06*	17:51:26.0	+24.34	+122.22	53	6.2
06/08/17*	11:11:35.0	+55.62	+161.69	55	5.7	07/09/10*	01:49:11.0	+2.97	-77.96	15	6.8
06/08/17*	15:20:35.0	+46.54	+141.91	14	5.6	07/09/12*	11:10:26.0	-4.44	+101.37	34	8.5
06/08/20*	03:01:02.0	+49.82	+156.41	26	6.0	07/09/12*	14:40:05.0	-3.16	+101.46	35	5.9
06/08/24*	21:50:36.0	+51.15	+157.52	43	6.5	07/09/12*	23:49:03.0	-2.62	+100.84	35	7.9
06/08/26*	23:46:18.0	+51.38	-179.54	35	5.7	07/09/13*	02:30:03.0	-1.69	+99.67	28	6.5
06/09/24*	22:56:21.0	-17.74	+41.81	6	5.7	07/09/13*	03:35:28.0	-2.13	+99.63	22	7.0
06/09/28*	01:36:48.0	+46.46	+153.36	11	5.9	07/09/13*	09:48:45.0	+3.80	+126.34	26	6.3
06/09/30*	17:50:23.0	+46.35	+153.17	11	6.6	07/09/13*	16:09:16.0	-3.17	+101.52	53	6.0
06/10/01*	09:06:02.0	+46.47	+153.24	19	6.5	07/09/14*	06:01:32.0	-4.07	+101.17	23	6.4
06/10/09*	10:01:47.0	+20.65	+120.02	14	6.3	07/09/20*	08:31:14.0	-2.00	+100.14	30	6.7
06/10/10*	23:58:04.0	+37.20	+142.66	9	6.0	07/09/28	13:38:57.0	+22.01	+142.67	260	7.5
06/10/23*	21:17:19.0	+29.35	+140.27	11	6.4	07/10/02*	03:43:38.0	-4.24	+101.21	22	5.9
06/11/15*	11:14:13.0	+46.59	+153.27	10	8.3	07/10/24*	21:02:50.0	-3.90	+101.02	21	6.8
06/12/01	03:58:21.0	+3.39	+99.08	204	6.3	07/10/25*	13:50:04.0	+46.01	+154.23	20	6.1
06/12/07*	19:10:21.0	+46.15	+154.39	16	6.4	07/11/16	03:13:00.0	-2.31	-77.84	122	6.8
06/12/26*	12:26:21.0	+21.80	+120.55	10	7.1	07/11/22*	23:02:12.0	+4.74	+95.06	49	5.8
06/12/26*	12:34:13.0	+21.97	+120.49	10	6.9	07/11/27*	04:26:58.0	+16.06	+119.84	35	5.9
06/12/30*	08:30:49.0	+13.31	+51.37	15	6.6	07/11/29	19:00:20.0	+14.94	-61.27	156	7.4
07/01/08*	17:21:49.0	+39.80	+70.31	16	6.0	07/12/19*	09:30:27.0	+51.36	-179.51	34	7.2
07/01/13*	04:23:21.0	+46.24	+154.52	10	8.1	07/12/26*	22:04:54.0	+52.56	-168.22	25	6.4
07/01/30*	21:37:44.0	+20.98	+144.71	20	6.6	08/01/09*	08:26:45.0	+32.29	+85.17	10	6.4
07/02/17*	00:02:56.0	+41.79	+143.55	31	6.0	08/01/22*	17:14:57.0	+1.01	+97.44	20	6.2
07/03/06*	03:49:38.0	-0.49	+100.50	19	6.4	08/02/08*	09:38:14.0	+10.67	-41.90	9	6.9
07/03/06*	05:49:25.0	-0.49	+100.53	11	6.3	08/02/20*	08:08:30.0	+2.77	+95.96	26	7.4
07/04/03	03:35:07.0	+36.45	+70.69	222	6.2	08/02/25*	08:36:33.0	-2.49	+99.97	25	7.2
07/04/07*	07:09:25.0	+37.31	-24.49	8	6.1	08/02/25*	18:06:03.0	-2.33	+99.89	25	6.6
07/04/27*	08:02:49.0	+5.36	+94.64	38	5.9	08/02/25*	21:02:18.0	-2.24	+99.81	25	6.7
07/05/04*	12:06:51.0	-1.41	-14.92	7	6.2	08/03/03*	14:11:14.0	+13.35	+125.63	24	6.9
07/05/05*	08:51:39.0	+34.25	+81.97	9	6.1	08/03/20*	22:32:57.0	+35.49	+81.47	10	7.2
07/05/16*	08:56:14.0	+20.50	+100.73	9	6.3	08/03/22	21:24:11.0	+52.18	-178.72	132	6.2
07/05/30	20:22:12.0	+52.14	+157.29	116	6.4	08/04/15*	22:59:51.0	+51.86	-179.36	11	6.4
07/06/02*	21:34:57.0	+23.03	+101.05	5	6.1	08/04/23*	18:28:41.0	+22.88	+121.62	10	6.0
07/07/01	04:12:07.0	+43.66	+144.73	130	5.8	08/05/02*	01:33:37.0	+51.86	-177.53	14	6.6
07/07/03*	08:26:00.0	+0.71	-30.27	10	6.3	08/05/07*	16:02:02.0	+36.18	+141.54	19	6.2
07/07/15*	13:08:01.0	+52.49	-168.04	15	6.1	08/05/13*	07:07:08.0	+30.89	+103.19	9	5.8
07/07/15*	13:26:15.0	+52.36	-168.01	10	5.9	08/05/17*	17:08:25.0	+32.24	+104.98	9	5.8
07/07/16*	01:13:22.0	+37.53	+138.45	12	6.6	08/05/20*	13:53:35.0	+51.16	+178.76	27	6.3
07/07/16	14:17:37.0	+36.81	+134.85	350	6.8	08/05/23*	19:35:34.0	+7.31	-34.90	8	6.5
07/07/20*	10:06:52.0	+42.91	+82.38	10	5.6	08/05/25*	08:21:49.0	+32.56	+105.42	18	6.1
07/07/25*	23:37:31.0	+7.16	+92.52	15	6.1	08/05/25*	19:18:25.0	+56.09	-153.78	22	6.0
07/07/26*	05:40:16.0	+2.87	+127.46	25	6.9	08/06/01*	01:57:23.0	+20.12	+121.35	31	6.3
07/07/29*	04:54:36.0	+53.64	+169.70	25	5.9	08/06/13*	23:43:45.0	+39.03	+140.88	7	6.9

07/07/30*	22:42:05.0	+19.31	+95.61	14	5.6	08/06/22*	23:56:30.0	+67.70	+141.28	18	6.1
07/07/31*	22:55:31.0	-0.16	-17.80	11	6.2	08/06/27*	11:40:13.0	+11.01	+91.82	17	6.6
07/08/02*	03:21:42.0	+51.31	-179.97	21	6.7	08/06/28*	12:54:46.0	+10.85	+91.71	15	6.1
07/08/07*	00:02:24.0	+27.29	+126.84	18	6.0	08/06/29	20:53:04.0	+45.16	+137.45	326	6.0
07/08/08	17:05:04.0	-5.86	+107.42	280	7.5	08/07/05	02:12:04.0	+53.88	+152.89	632	7.7
07/08/15*	20:22:11.0	+50.32	-177.55	9	6.5	08/07/06*	01:00:08.0	+45.35	+151.04	22	5.7
07/08/20*	13:46:17.0	+6.13	+127.38	8	6.4	08/07/06*	09:08:21.0	+45.39	+150.96	17	5.7
07/08/20*	22:42:28.0	+8.04	-39.25	6	6.5	08/07/13*	14:58:33.0	+21.01	+121.15	14	6.2

Table S2 Parameters of 2.5D models of the structure beneath the seismic stations.

PA73						SUW					
Depth [km]	Vp [km/s]	Vs [km/s]	Density [g/cm^3]	Strike [^{o}]	Dip [^{o}]	Depth [km]	Vp [km/s]	Vs [km/s]	Density [g/cm^3]	Strike [^{o}]	Dip [^{o}]
0.00	1.87	1.04	2.11	72.10	0.49	0.00	1.86	1.04	2.11	67.7	0.3
0.20	2.30	1.28	2.17	110.34	0.31	0.27	2.32	1.29	2.18	92.9	0.4
0.42	2.42	1.34	2.19	116.63	0.33	0.42	2.38	1.32	2.18	153.9	0.2
0.56	2.44	1.36	2.19	117.54	0.41	0.53	2.35	1.30	2.18	158.3	0.2
0.80	4.38	2.43	2.46	113.02	0.59	0.58	3.92	2.18	2.40	170.9	3.3
0.84	4.06	2.26	2.42	103.61	1.49	0.70	6.20	3.71	2.79	222.3	0.7
1.32	6.34	3.79	2.82	223.20	0.82	16.02	6.59	3.81	2.89	38.4	2.5
15.15	6.56	3.79	2.88	47.73	2.07	26.96	6.98	3.94	3.01	63.8	4.0
25.38	6.97	3.94	3.00	111.80	2.77	46.31	8.27	4.59	3.42	-34.8	2.5
46.67	8.27	4.59	3.42	-36.46	2.70						

PQ47						GKP					
Depth [km]	Vp [km/s]	Vs [km/s]	Density [g/cm^3]	Strike [^{o}]	Dip [^{o}]	Depth [km]	Vp [km/s]	Vs [km/s]	Density [g/cm^3]	Strike [^{o}]	Dip [^{o}]
0.00	1.84	1.03	2.11	78.6	0.4	0.00	1.85	1.03	2.12	75.3	0.3
0.23	3.11	1.73	2.29	72.7	0.2	0.18	2.54	1.41	2.21	67.4	0.1
1.46	3.84	2.13	2.39	58.7	3.0	0.48	3.57	1.98	2.35	17.7	3.5
1.59	4.38	2.43	2.46	49.9	3.0	2.27	4.95	2.75	2.55	56.9	4.1
3.31	5.07	2.81	2.56	60.3	3.3	4.96	5.44	3.02	2.62	88.5	2.5
5.21	5.19	2.88	2.58	-6.5	2.7	12.28	6.02	3.60	2.75	112.0	5.2
10.23	5.91	3.54	2.72	-2.3	4.8	18.01	6.58	3.80	2.88	91.9	3.1
20.25	6.49	3.75	2.86	75.2	2.9	28.13	7.06	3.99	3.03	29.5	3.9
25.74	7.15	4.04	3.06	7.8	2.2	38.95	8.36	4.64	3.44	4.8	5.5
32.48	8.35	4.64	3.44	-5.0	3.7						

PG42						KSP					
Depth [km]	Vp [km/s]	Vs [km/s]	Density [g/cm^3]	Strike [^{o}]	Dip [^{o}]	Depth [km]	Vp [km/s]	Vs [km/s]	Density [g/cm^3]	Strike [^{o}]	Dip [^{o}]
0.00	1.80	1.00	2.10	83.1	0.3	0.00	4.44	2.47	2.47	119.8	4.4
0.04	3.40	1.89	2.33	9.5	1.2	1.27	6.05	3.63	2.75	96.3	1.7
1.14	4.73	2.63	2.51	39.9	1.2	16.35	6.24	3.61	2.80	22.1	2.6
1.16	4.88	2.71	2.54	41.1	2.9	22.26	6.76	3.82	2.94	42.8	1.1
3.69	5.99	3.59	2.74	41.6	1.9	34.09	8.02	4.46	3.34	54.0	4.8
13.21	6.28	3.63	2.81	132.9	4.1						
24.41	6.77	3.83	2.94	28.5	3.4						
30.99	8.15	4.53	3.38	100.1	1.7						

Fig. S1 Example of the rotation of seismograms recorded by the analyzed seismic stations for an earthquake in the Andreanof Islands with back azimuth angles found by the RF-rotation procedure. RFR and RFT are shown too. Seismograms are filtered with a band-pass Butterworth filter of corner frequencies 0.03 and 1 Hz. The amplitude scales are different for seismograms and RFs. Time 0 s refers to the theoretical P-onset calculated from the *iasp91* model. Delay time zero refers to the direct P wave.

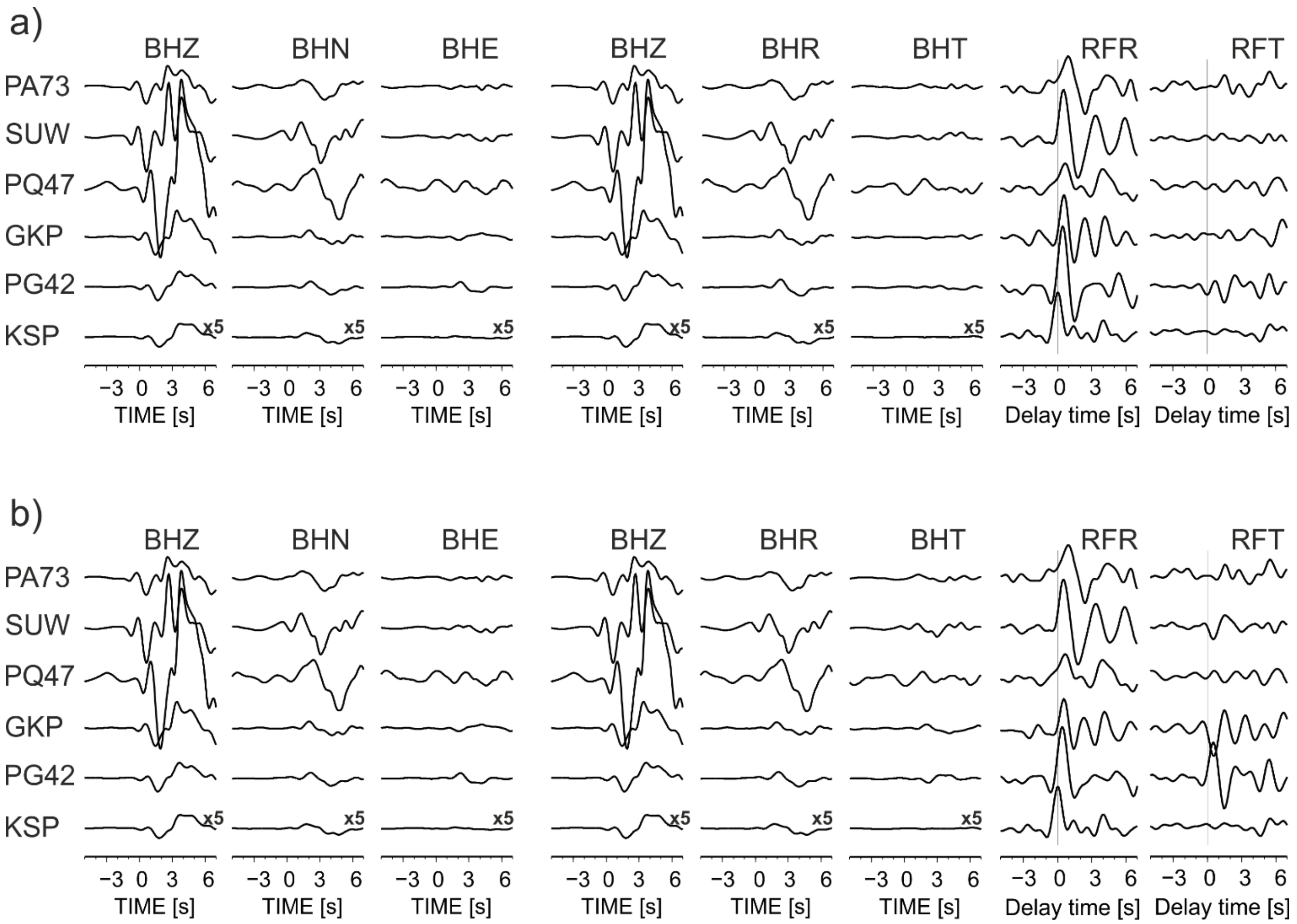

Fig. S2 Example of the rotation of seismograms recorded by the analyzed seismic stations for an earthquake in the Andreanof Islands with polarization angles found by the RF-rotation procedure. RFQ and RFT are also shown. Seismograms are filtered with a band-pass Butterworth filter of corner frequencies 0.03 and 1 Hz. The amplitude scales are different for seismograms and RFs. Time 0 s refers to the theoretical P-onset calculated from the *iasp91* model. Delay time zero refers to the direct P wave.

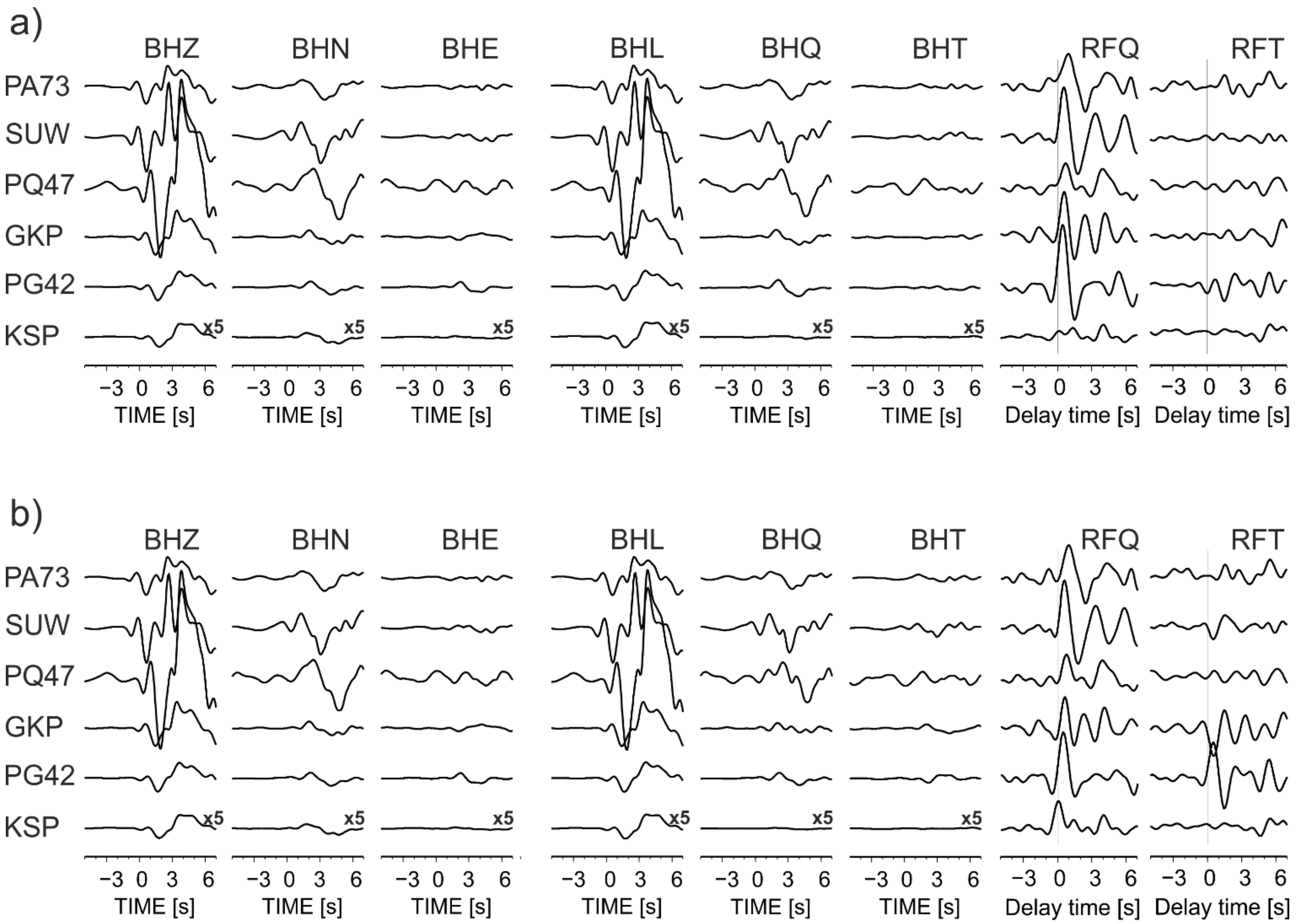

Fig. S3 Orientation of a station's sensor calculated as the difference between the theoretical and observed back azimuth angles based on the RF-rotation procedure (RF) and Rayleigh wave polarization analysis (Rayleigh).

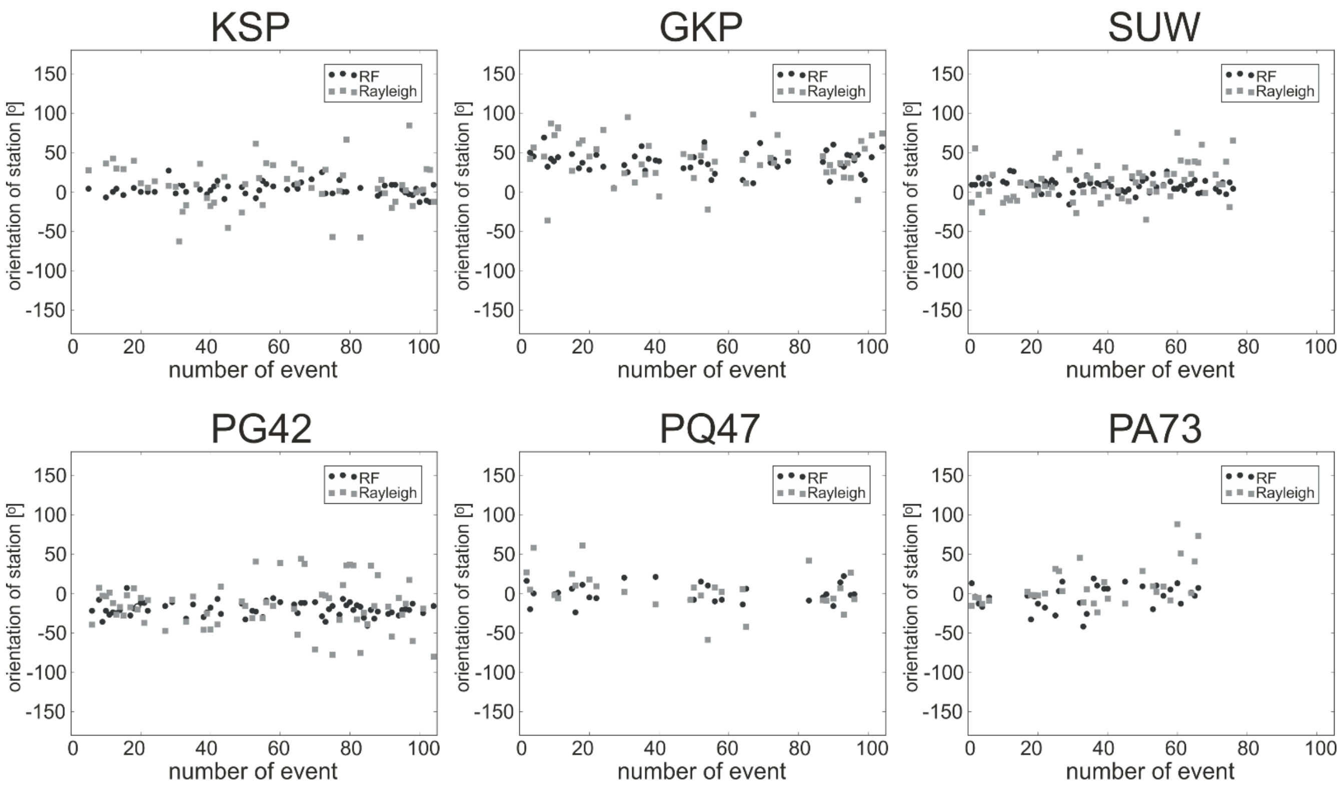

Fig. S4 Back-azimuthal distribution of theoretical and calculated from the RF-rotation procedure (observed) polarization angles

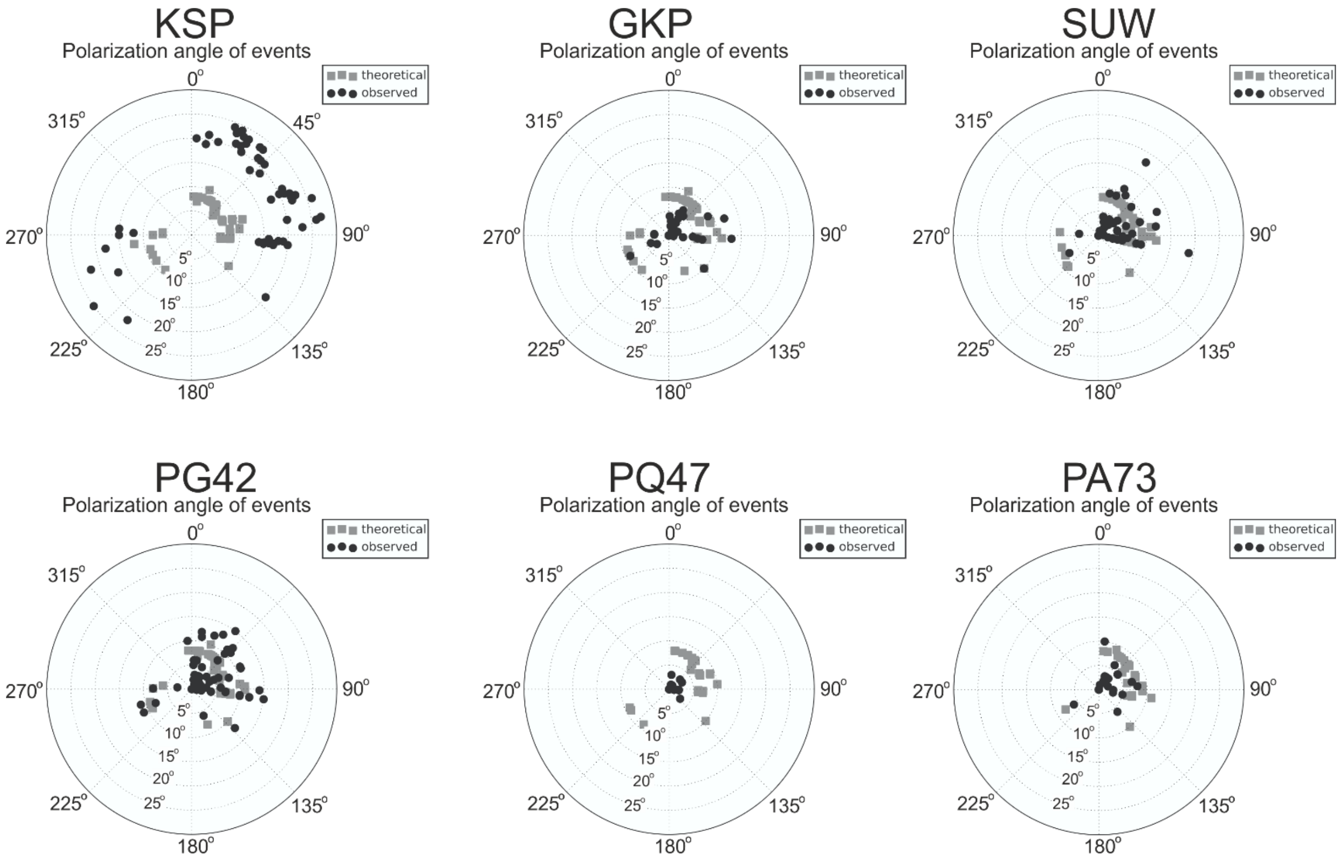

Fig. S5 Stacked RF for the analyzed seismic stations, sorted versus the theoretical back azimuth of events. RFs calculated based on the RF-rotation procedure are marked by solid black lines and shaded areas; RFs calculated by the modified ray-tracing method for 2.5D models (see Table S2) of the structure beneath each station are marked by grey lines. RFs are filtered with a low-pass Butterworth filter of corner frequency 0.8 Hz. The amplitude scale is the same for all components. Delay time zero refers to the direct P wave.

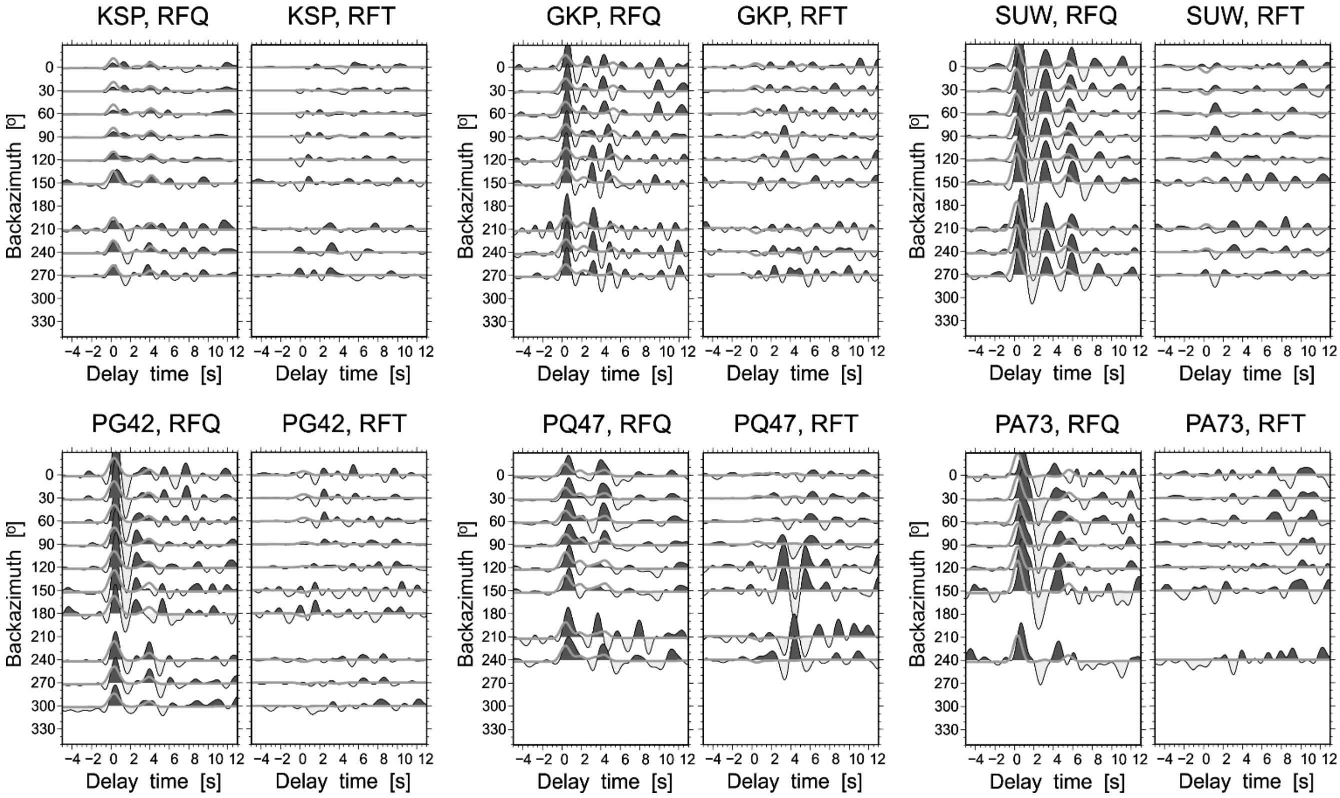

Supplement: Supplementary file 1 — (PDF 1544 kb) [file 10950_2017_9640_MOESM1_ESM.pdf]
